# First high-quality genome assembly data of sago palm (*Metroxylon sagu* Rottboll)

**DOI:** 10.1016/j.dib.2022.107800

**Published:** 2022-01-06

**Authors:** Leonard Whye Kit Lim, Melinda Mei Lin Lau, Hung Hui Chung, Hasnain Hussain, Han Ming Gan

**Affiliations:** a Faculty of Resource Science and Technology, Universiti Malaysia Sarawak, 94300 Kota Samarahan, Sarawak, Malaysia; b Centre for Sago Research (CoSAR), Faculty of Resource Science and Technology, Universiti Malaysia Sarawak, 94300 Kota Samarahan, Sarawak, Malaysia; c GeneSEQ Sdn Bhd, Bukit Beruntung, 48300 Rawang, Selangor, Malaysia; d Centre for Integrative Ecology, School of Life and Environmental Sciences, Deakin University, Geelong, Victoria, Australia

**Keywords:** aa, amino acid, bp, base pair, BUSCO, Benchmarking Universal Single Copy Orthologs, KEGG, Kyoto Encyclopedia of Genes and Genomes, SDS, Sodium dodecyl sulfate, *Metroxylon sagu*, Sago palm, Genome annotation, Starch synthesis

## Abstract

The sago palm (*Metroxylon sagu* Rottboll) is a tropical halophytic starch-producing, economically important crop palm mainly located in Southeast Asian countries. Recently, a genome survey was conducted on this palm using the Illumina sequencing platform, with a very low (21.5%) BUSCO genome completeness score, and most of them (∼78%) are either fragmented or missing. Thus, in this study, the sago palm genome completeness was further improved with the utilization of the Nanopore sequencing platform that produced longer reads. A hybrid genome assembly was conducted, and the outcome was a much complete sago palm genome with BUSCO completeness achieved at as high as 97.9%, with only ∼2% of them either fragmented or missing. The estimated genome size of the sago palm is 509,812,790 bp in this study. A sum of 33,242 protein-coding genes was revealed from the sago palm genome and around 96.39% of them had been functionally annotated. An investigation on the carbohydrate metabolism KEGG pathways also unearthed that starch synthesis was one of the major sago palm activities. The genome data obtained from this work is indispensable for future molecular evolutionary and genome-wide association studies on the economically important sago palm.

## Specifications Table

**Table:** 

Subject	Biological Sciences
Specific subject area	Genomics
Type of data	Sequencing raw reads, Table and Figure
How data were acquired	Sequencing
Data format	Raw Reads (fastq), Assembly (fasta)
Parameters for data collection	Total DNA was collected from *Metroxylon sagu* planted in the living germplasm of Universiti Malaysia Sarawak East Campus (1°27′52.3″N, 110°27′04.7″E).
Description of data collection	About 5 µg of unfragmented genomic DNA libraries were prepared using LSK110 kit according to the manufacturer's protocols. The resultant libraries were sequenced on the Oxford Nanopore PromethION platform. Reads were basecalled employing high-accuracy mode in Guppy version 4, and only passed reads were uploaded to the NCBI SRA database. The minimum cutoff length was set at 500 bp.
Data source location	Institution: Universiti Malaysia Sarawak City/Town/Region: Kota Samarahan, Sarawak, Malaysia Latitude and longitude for collected samples/data: (1°27′52.3″N, 110°27′04.7″E).
Data accessibility	With the article. The sequencing reads used in assembly analysis of *Metroxylon sagu* are available under the NCBI BioProject PRJNA769197 (https://www.ncbi.nlm.nih.gov/bioproject/769197)

## Value of the Data

• First complete genome dataset for the eco-economic important sago palm (*Metroxylon sagu* Rottboll).
• High completeness of the sago palm genomic dataset will facilitate future research, such as genome-wide association studies.
• The data is useful in pioneering sago palm genetic landscape investigations which in turn unmask the mystery behind its high starch yield, salinity tolerance and disease resistance.

## 1 Data Description

The sago palm genome was estimated at 509,812,790 bp in this study. The sago palm genome sequencing from this study had greatly improved the genome completeness in an unprecedented manner. A previous report on BUSCO genome completeness of sago palm by [1] revealed 21.5% single-copy complete genes and 1.1% duplicated complete genes, whereas 32.2% and 45.2% are fragmented and missing, respectively. In this study, the nanopore approach coupled with the Illumina method has yielded 97.9% complete genes (89.8% single-copy and 8.1% duplicated), 1.1% is fragmented, and only 1% is missing (Table 1). The total number of contigs in this study is 2025, which is minuscule compared to the 739,583,200 contigs reported on the sago genome survey [1]. However, the overall contig lengths in this study is higher than that reported by [1], with 1801 contigs documented in this study to have at least 50,000 bp length in contrast to the Illumina short sequencing reads whereby majority of them are 100 to 2000 bp long [1]. On a side note, the GC content of this improved sago palm genome assembly is 36.5%, which is a little lower than that reported by [1] (37.31%).

**Table 1:** Genome statistics of the sago palm (*Metroxylon sagu* Rottboll) genome.

Organism	*Metroxylon sagu*
SRA	SUB10461460
Bioproject	PRJNA769197
Biosample	SAMN15953627
Accession number	JACVBY000000000
BUSCO completeness (Total BUSCOs used as references from the embryophyta_odb10 database: 1614)	97.9% complete (89.8% single-copy; 8.1% duplicated); 1.1% fragmented, 1% missing
# contigs (>= 0 bp)	2025
# contigs (>= 1000 bp)	2025
# contigs (>= 5000 bp)	2024
# contigs (>= 10,000 bp)	2022
# contigs (>= 25,000 bp)	2002
# contigs (>= 50,000 bp)	1801
Total length (>= 0 bp)	509,812,790 bp
Total length (>= 1000 bp)	509,812,790 bp
Total length (>= 5000 bp)	509,810,926 bp
Total length (>= 10,000 bp)	509,794,905 bp
Total length (>= 25,000 bp)	509,389,047 bp
Total length (>= 50,000 bp)	501,302,081 bp
# contigs	2025
Largest contig	4,418,102 bp
Genome size	509,812,790 bp
GC (%)	36.50
N50	453,371 bp
N75	220,193 bp
L50	341 bp
L75	742 bp
# N's per 100 kbp	0.00
**Genome annotation**	
# protein-coding genes	33,242
# functionally annotated proteins	32,041
Mean protein length	313 aa
Longest protein	3768 aa (titin protein)
Shortest protein	23 aa (TIGR01906 family membrane protein)
Mean exon length	276 bp
Longest exon	8579 bp (heavy metal-associated isoprenylated plant protein 3)
Shortest exon	2 bp (uncharacterized gene)
Mean intron length	926 bp
Longest intron	25,685 bp (putative eukaryotic translation initiation factor)
Shortest intron	5 bp (pyruvase kinase isozyme A)

A total of 33,242 protein-coding genes were discovered from the sago palm genome in this study. Around 96.39% (32,041) of these protein-coding genes have been successfully annotated in terms of functionality. The mean protein length reported in this study is 313 aa. The largest protein found within the sago palm genome in this study is the titin protein with an amino acid length of 3768 aa whereas the smallest protein is only 23 aa, namely the TIGR01906 family membrane protein. The mean exon and intron lengths uncovered in this study are 276 bp and 926 bp correspondingly. The lengthiest exon is 8579 bp long, revealed within the heavy metal-associated isoprenylated plant protein 3 gene. An uncharacterized sago palm gene contains the shortest exon of 2 bp among all other exons within the genome. The longest sago palm genome intron belongs to that of the putative eukaryotic translation initiation factor gene, with 25,685 bp in length. The shortest intron (5 bp) was unearthed within the pyruvase kinase isozyme A gene of the sago palm.

**Fig. 1:**
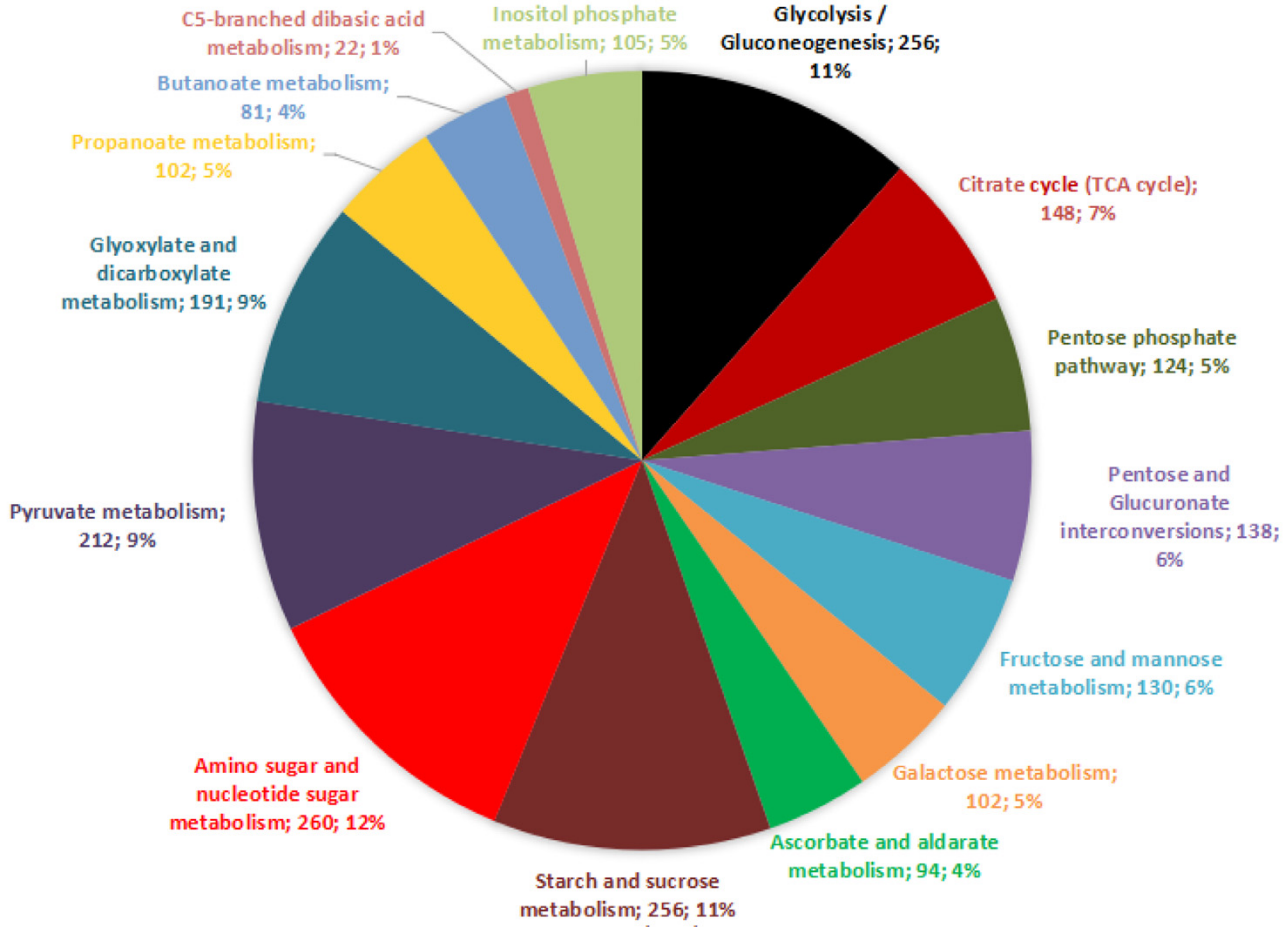
The breakdown of the KEGG carbohydrate metabolism pathways the sago palm genes are involved in.

All protein-coding genes of sago palm were subjected to functional annotation. The focus was placed on the carbohydrate metabolism KEGG category as this is one of the most imperative yet untapped aspects of the sago palm, contributing to its high starch yield. Under the carbohydrate metabolism category, there are 15 metabolic pathways described, namely glycolysis/gluconeogenesis, citrate cycle (TCA cycle), pentose phosphate pathway, pentose and glucuronate interconversions, fructose and mannose metabolism, galactose metabolism, ascorbate and aldarate metabolism, starch and sucrose metabolism, amino sugar and nucleotide sugar metabolism, pyruvate metabolism, glyoxylate and dicarboxylate metabolism, propanoate metabolism, butanoate metabolism, C5-branched dibasic acid metabolism, as well as inositol phosphate metabolism. A total of 2221 protein-coding genes were unveiled to be closely related to the 15 aforementioned carbohydrate metabolism pathways (Fig. 1). Majority of them (260 or 12%) are related to amino sugar and nucleotide sugar metabolism. Almost an equal number of genes (256 or 11%) were associated with glycolysis/gluconeogenesis and starch and sucrose metabolism pathways. Only 1% (22) of them are involved in the C5-branched dibasic acid metabolism. This phenomenon shows that starch production is the major limelight among all other carbohydrate metabolism pathways in the sago palm genome context [2,3].

**Fig. 2:**
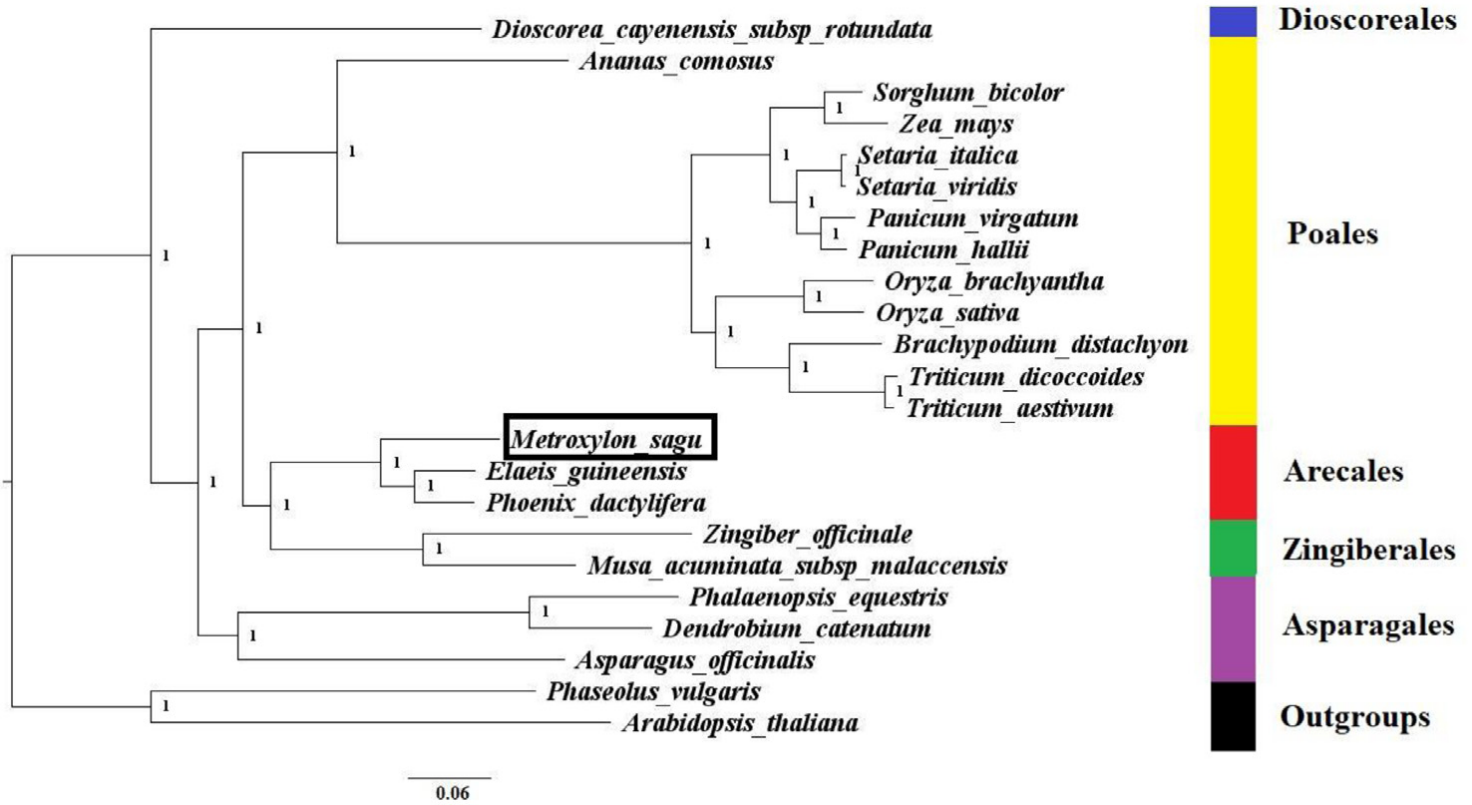
The maximum likelihood whole genome phylogenetic tree constructed based on BUSCO genes across all the available full monocot genomes (with complete records of coding sequences and proteins) found within the GenBank public database, with 1000 bootstrap replications. The species targeted in this study were highlighted in a black box.

To date, there are only 20 representative full monocot genomes with complete records of coding sequences and proteins found within the GenBank public database. The maximum likelihood phylogenetic tree was constructed based on single-copy proteins across the full genomes of 21 monocots (including sago palm) and two dicot outgroups (Fig. 2). Five major clades were found within the tree plotted and each of them represents an order, namely Dioscoreales, Poales, Arecales, Zingiberales, and Asparagales. Most clades are backed with strong bootstrap ratio of 1, indicating high bootstrap confidence. In this study, the sago palm was grouped under the order Arecales along with two other palm members, the oil and date palm. The Arecales order was underrepresented due to the lack of available full genome sequences [4], [5], [6]. More informative branching can be observed among the palm members when more full genomes are sequenced in the future as seen in the sago palm phylogenetic trees constructed based on chloroplast genome and ITS2 region [1,7]. In a nutshell, this sago palm complete genome would be a valuable resource for future molecular evolutionary studies as well as genome-wide association studies.

## 2 Experimental Design, Materials and Methods

### 2.1 Whole genome sequencing

Fresh sago palm leaves were harvested from the tree chosen by [6] for whole chloroplast sequencing in which this tree has been previously verified by our plant expert team from the living germplasm of Universiti Malaysia Sarawak East Campus (1°27′52.3″N, 110°27′04.7″E). The Illumina reads of sago palm were obtained from [1] which was sequenced using Illumina HiSeq X.

In order to produce Oxford Nanopore long reads, the genomic DNA was first extracted from fresh sago palm leaves using high-salt SDS approach. About 5 µg of unfragmented genomic DNA libraries were prepared using LSK110 kit according to manufacturer's protocols. The resultant libraries were sequenced on Oxford Nanopore PromethION platform. Reads were basecalled employing high-accuracy mode in Guppy version 4 and only passed reads were uploaded to the NCBI SRA database. The minimum cutoff length was set at 500 bp.

### 2.2 Hybrid genome assembly and genome annotation

The Maryland Super-Read Celera Assembler v.3.2.2 was utilized to perform the *de novo* hybrid genome assembly of the sago palm. The sago palm hybrid genome assembly was conducted emulating that of [8,9].

The prediction of all protein-coding genes was conducted using Augustus. A BUSCO analysis was performed on the annotated sago palm genome dataset utilizing the embryophyta-specific dataset as reference. TransDecoder v5.5.0 was employed to extract the protein coding sequences prior to the clustering at 98% similarity of protein using cdhit v4.7 (-g 1 -c 98). The functional annotation was done using eggNOGmappr (evolutionary genealogy of genes: Non-supervised Orthologous Groups) with a 0.001 minimum E-value.

### 2.3 Phylogenetic analysis

All available full monocot genomes (with complete records of coding sequences and proteins) found within the GenBank public database were downloaded, totaling to 20 monocots and two dicot outgroups (only one representative was selected for each duplicated species). BUSCO analysis was conducted to all full genomes downloaded to extract all single-copy proteins. These proteins were aligned using Muscle prior to the maximum likelihood phylogenetic tree plotting using iqtree with 1000 bootstrap replications. The tree was visually improved using FigTree software.

## Ethics Statement

The collection of plant material was carried out in strict accordance with the guidelines outlined by the germplasm committees of Universiti Malaysia Sarawak, Malaysia.

## CRediT authorship contribution statement

**Leonard Whye Kit Lim:** Data curation, Writing – original draft. **Melinda Mei Lin Lau:** Data curation. **Hung Hui Chung:** Conceptualization, Funding acquisition, Writing – review & editing. **Hasnain Hussain:** Writing – review & editing. **Han Ming Gan:** Methodology, Conceptualization, Writing – review & editing.

## Declaration of Competing Interest

The authors declare that they have no known competing financial interests or personal relationships which have or could be perceived to have influenced the work reported in this article.
